# Monthly Summary

**Published:** 1883-09

**Authors:** 


### Monthly Summary.

Successful Exsection of Inferior Dental Nerve for Neuralgia; Bone Grafting.—Mr. M., aged 60, consulted me on Nov. 9th, 1882, for persistent pain on the alveolar border of the lower jaw (right side) midway between the angle and the symphysis. The pain had been present for five years, with exacerbations which rendered his life unbearabie. There were no teeth in the lower jaw except the incisors. I proposed to him to cut down and remove a piece of the inferior dental nerve, and to this he willingly assented. On the next day, however, I tried the effect of dividing the inferior dental nerve at its entrance into the inferior dental foramen. As this failed to give any relief, I made an incision about two inches long, parallel to the lower border of the jaw, dividing the facial artery, which I tied. I then divided the periosteum, and separated it sufficiently from the bone to allow of the application of the trephine. A trephine, half an inch in diameter, was then applied over the site of the nerve, and after cutting down about half an inch, the bone was elevated, and a slight additional application of the instrument opened the inferior dental canal, where the nerve was seen lying. I then removed half an inch of the nerve, and at the suggestion of Drs. Stirling and Jay, I replaced the plug of bone to try an experiment in bone-grafting, and drew the divided edges of the periosteum over it. The operation was followed by complete relief to all the symptoms, and the wound was perfectly healed in a week, with the exception of a small opening where one of the ligatures came out. The plug which was replaced has not caused the slightest irritation, and may now reasonably be supposed to have become reunited to the rest of the bone.—*W Gardner, M. D. in Australian Medical Journal.*

HOT WATER A RESTORATIVE IN CHLOROFORM NARCOSIS.—Dr. A. Holmes of Clinton, North Carolina, writes as follows to the *North Carolina Medical Journal :*

I will call attention to a remedy with which I have been quite successful in several cases, and am satisfied it is a most valuble agent. The application of as *hot water* as can be borne without injury to the parts, in cverdosing with chloroform, or where patients are easily impressed and there is danger to life. My mode of using, is to dip folded cloths or towels in the water, *and I repeat hot water*, and apply to the head, and so continue until reaction is established

I have used it several times, and only a few weeks ago whilst amputating a leg, the patient was rapidly sinking from the effects of chloroform, (and being an old man probably from the shock and loss of blood) I used diligently the ordinary means for restoration, such as lowering the head, drawing forward the tongue, pressure upon the chest, friction, warmth, ammonia to the nostril, &c., and for a time I feared death was inevitable. Hot water being convenient, its application was made as above stated, and almost instantly there was movement of the head and extremities, and in a short time restoration was established, and my patient soon recovered.

You and others may be familiar with the use of *hot water* in chloroform poisoning ; but I have never heard of its being used in this way before, and my friend, Dr Stevens, a recent graduate of the Jefferson Medical College, who assisted in the above operation, said it was not used in the hospitals or clinics and was so much pleased with its action that he insisted it should be given to the profession.

I ascribe the good effect to the shock and warmth, causing a rapid return of blood to the brain. It is certainly a safe, convenient, and in my experience, a valuable agent, and I sincerely trust others may find it, if not the remedy so much needed, a potent adjuvant,

---

SYPHILIS AND RACHITIS.—M. Parrot read before the Academie de Medecine his researches upon this subject, and he has come to the conclusion that rachitis is the special lesion which hereditary syphilis produces in the bone. He further states that in all cases of hereditary syphilis there are found, from the last months of intra-uterine life to a period near the second dentition, systematic, polymorphous alterations of the skeleton, and all of them resemble lesions of rachitis. Several patients were presented by M. Parrot to the Society, affected with syphilitic osseous lesions, varying in degree, all of which would have been formerly considered rachitie lesions.

At a later meeting of the Society, M. Cazin presented a communication upon this subject, in which he opposed the views of Parrot, and mentioned numerous cases in which rachitis was manifest without it being possible to discover any trace of a syphilitic history. The writer ended his paper by saying, that if we did not accurately know the nature of rachitis, this affection is certainly not a metamorphosis of syphilis. M. Lucas-Champlonniere supported the views of M. Cazin, and said he had never considered syphilis as a cause of rachitis. M. Despres did not accept the opinion of M. Parrot, as to the relation of rachitis and syphilis; the latter, he said, had been able to base his opinion, up to a certain point, upon the pathalgical anatomy of the affections, but their clinical nature absolutely opposed the idea of their identity.—*Gazette Med. de Paris.*

---

INODOROUS IODOFORM.—The peculiar odor of iodoform is found to be well masked by the addition of attar of rose, one minim to the drachm, or of essence of rose geranium, three or four minims to the drachm. The clinic room gets to smell like a flourist's shop.—*Polyclinic.*

---

NEW DISCOVERIES, especially in anatomy are not always novel. The so-called "supplementary anterior ventricle of the larynx" is simply the *fovea centrale* of Merkel, which is described in our best text-books on diseases of the throat. It is a little foramen at the anterior insertion of the vocal bands, leading into the ventricles upon either side. It exists much more pronounced in some of the lower animals, and in man is merely rudimeotary.—*Polyclinic.*

